# Pharmacokinetics of Conventional and Long-Acting Oxytetracycline Preparations in Kilis Goat

**DOI:** 10.3389/fvets.2017.00229

**Published:** 2017-12-22

**Authors:** İbrahim Aktas, Ender Yarsan

**Affiliations:** ^1^Faculty of Veterinary Medicine, Department of Pharmacology and Toxicology, Ankara University, Ankara, Turkey

**Keywords:** pharmacokinetics, Kilis goat, oxytetracycline, long-acting, conventional

## Abstract

The pharmacokinetics of conventional and long-acting (LA) oxytetracycline (OTC), widely used broad-spectrum antibacterial drugs in veterinary medicine, were evaluated in Kilis goats at single dosage of 20 mg/kg body weight (bw). A total of 21 goats were divided into three groups: intravenous (Group I) and intramuscular (IM) (Group II) administration of the conventional formulation and IM administration of the LA formulation (Group III). Blood samples were taken at 0.25, 0.5, 1, 2, 4, 8, 12, 24, 36, 48, 60, 72, and 96 h; and OTC analysis was performed by HPLC. For Group III and Group II, time to reach maximal plasma drug concentration (*T*_max_) was 0.6 ± 0.28 and 0.46 ± 0.09 h and maximal plasma drug concentrations (*C*_max_) were 8.72 ± 2.47 μg/ml and 13.57 ± 5.83 μg/ml, respectively. In Group I, *C*_0_ concentration was found to be 63.51 ± 11.59 μg/ml. The elimination times (*T*_1/2_) were 10.84 ± 3.20, 27.96 ± 11.66, and 10.47 ± 1.30 h; and AUC were 115 ± 29.12, 96.44 ± 9.49, and 80.86 ± 12.76 μg/ml/h for Group I; Group II, and Group III, respectively. Bioavailability by IM administration were 69.71% for the conventional OTC and 83.15% for the LA OTC.

## Introduction

Kilis Goat is a hybrid of hair goat and goat of Aleppo. The hair is usually black, but can also be brown, tan, gray, or tawny. As a trait, they are large with a long body; where the females have a developed mammary gland along with good dairy performance (1), corresponding to the highest milk yield among the other domestic breeds. Production of the Kilis Goats is officially carried out in Gaziantep, Hatay, and particularly in Kilis. It is estimated that their number is between 60,000 and 70,000 (2, 3).

Oxytetracycline (OTC) is a broad-spectrum antibiotic, frequently used in veterinary medicine for gastrointestinal and respiratory system diseases, for mainly aerobic microorganisms including Gram-positive/negative bacteria, *Rickettsia, Mycoplasma*, and *Chlamydia* species. Long-acting (LA) formulation of the drug is available in different solvent. Those formulations are used considering a dose interval of 3 to 5 days. Furthermore, OTC, as an HCl salt, can be administered by different routes. It can be easily absorbed by the mammalian intestine, while the absorption is limited in poultry. After oral administration in ruminants, OTC loses its effect because of the front gut flora. Following oral administration, the value of *t*_1/2_ was previously described as 3.6 h in sheep and goats (4). Therefore, the parenteral route is preferred for the systemic effect (5). OTC, passes into intracellular fluid quickly when administered systemically. The drug can also pass the blood–brain barrier and the placenta as well (6). It accumulates in the internal organs, including liver, kidney, spleen, and the lungs, and also in developing parts of the bones (4). It passes to the eyes, synovial fluid, milk, and eggs (4). OTC is metabolized in the liver, where it is converted into tetracycline and then it is mostly excreted through the bile and urine (7–10). OTC is removed from the body by being filtered by glomerulae of the kidney. However, it remains long in the body when dispersed in excess proportion to the tissues and gets into circulation of the liver–intestine (11–14).

Antimicrobial agents are classified according to their pattern of antimicrobial activity and successful pharmacotherapy should integrate both the pharmacokinetic and the pharmacodynamic properties of antimicrobial agents (e.g., MIC, AUC, PAE). For this purpose, long-term formulations have been developed (4, 15). OTC is delivered *via* intramuscular (IM) and intravenous (IV) routes to sheep and goats at 2–10 mg/kg as a daily dose. LA preparations are administered to cattle, sheep, and goats at 20 mg/kg for 2–4 day intervals or 30 mg/kg for six daily intervals. LA formulations can provide adequate and well controlled level of drug for the systemic treatment or control for 3–4 days (16–19). These formulations are used in the treatment of acute diseases as well as chronic diseases (20). The aim of this study was to investigate the pharmacokinetic parameters of OTC in Kilis Goats, after IM and IV administration of conventional and LA formulations.

## Materials and Methods

Oxytetracycline preparations were used conventional (OTC hydrochloride salt 0.1 g/ml) and LA (OTC dihydrate salt preparations of milliliters of 0.2 g) formulations. Twenty-one Kilis male goats, around 12–13 months and 20–40 kg weight range were used in this study, and the parasitic load was previously tested. The animals were allowed to acclimate for a week before the study. Animals were given access to water *ad libitum*. Feed (without antibiotics contributes to dry weed, straw, and barley) requirements was met, twice per day. Animals were numbered using spray paint for a clear distinguishment. To perform the pharmacokinetic studies, the animals were divided into three groups of seven animals. Compliance with the principles of operation of the ethics committee was approved by the Ministry of Food Agriculture and Livestock Adana Veterinary Control Institute Ethics Board of the date 22.05.2014 and decision No. 29.

Group I, received 20 mg/kg *via* vena jugularis by IV route; Group 2 received the same dose by IM route through administration at musculus semitendinosus at 100 mg/ml concentrated formulation. Group 3 received LA formulation at 200 mg/ml at the same dose as 20 mg/kg by IM route.

Blood samples were taken and transferred to Ca EDTA tubes at 0.25, 0.5, 1, 2, 4, 8, 12, 24, 36, 48, 60, 72, and 96 h after drug administration. Samples were kept refrigerated until they arrived to the laboratory. Tubes were centrifuged (Hettich U320R) at 5,000 rpm for 15 min on the same day, and the plasma were separated to the labeled plastic tubes. For plasma samples, the method of Chamberlain (21) was adopted for the HPLC analysis of OTC determination. The mobile phase consisted of acetonitrile and oxalic acid (20:80 v/v) and was delivered (Thermo Finnigan Surveyor HPLC) at a flow rate of 0.7 ml/min. A nucleosil C_18_ analytical column (5 µm, 150 mm × 4.6 mm, Phenomenex synergi Max RP) was used with photo diode array detection at an excitation wavelength of 365 nm.

Pharmacokinetic parameters were calculated using the individual plasma concentration–time curve graphs plotted pharmacokinetic software program (WinNonlin, 5.0, Pharsight, USA). The peak plasma concentration (*C*_max_) and the time to reach the peak plasma concentration (*T*_max_) were determined from the concentration–time curve for each animal. The area under the plasma concentration–time curve (AUC) and the area under the first-torque curve was calculated by the trapezoidal method, the average length of stay. OTC bioavailability was calculated after administration intramuscularly calculated AUC and the ratio of the calculated AUC after IV route, using the following formula (%)*F* = (AUC_IM_/AUC_IV_) × 100.

The average pharmacokinetic parameters were evaluated by comparing the differences between OTC IV, OTC LA IM, and OTC IM Groups by Duncan test using a statistically one-way analysis of variance. The average value under *p* < 0.05 was considered as statistically different.

## Results

Retention time were found as 6.4 min; with a limit of detection (LOD) as 0.0125 g/ml and LOD as 0.025 µg/ml. Average recovery rate was 91.12%. The average plotted regression line for OTC concentration standards were calculated from 0.0125, 0.025, 0.05, 0.1, 0.2, 0.4, 0.8, 1.6, 3.2, 6.4, 12.8, 25, and 50 µg/ml concentrations with correlation coefficient as *r*^2^ = 0.9964 using *y* = 49,239*x* formula.

Kinetic parameters are shown in Table 1 for Groups 1, 2, and 3.

**Table 1:** Pharmacokinetic parameters of OTC in Kilis Goat following administration of conventional and long-acting formulations at a single dose of 20 mg/kg.

Pharmacokinetic parameters	OTC intra venous	OTC (LA) intra muscular	OTC intra muscular
*T*_1/2λz_ (h)	10.84 ± 3.20^a^ (7.75–17.36)	27.96 ± 11.66^b^ (20.76–54.09)	10.47 ± 1.30^a^ (8.36–12.33)
*T*_max_ (h)	–	0.60 ± 0.28^b^ (0.25–1)	0.46 ± 0.09^a^ (0.25–0.50)
*C*_max_ (μg/ml)	–	8.72 ± 2.47 (5.99–13.11)	13.57 ± 5.83 (5.93–21.01)
*T*_last_ (h)	58.28 ± 4.53^b^ (48–60)	89.14 ± 11.71^a^ (96–72)	70.29 ± 4.54^c^ (60–72)
*C_0_* (μg/ml)	63.51 ± 11.59 (45.70–78.64)	–	–
*C*_last_ (μg/ml)	0.04 ± 0.16^a^ (0.2–0.7)	0.23 ± 0.08^b^ (0.14–0.34)	0.04 ± 0.12^a^ (0.03–0.06)
AUC (μg/ml/h)	115.37 ± 29.22^b^ (65.17–154.99)	87.35 ± 10.91^a^ (68.81–99.73)	80.21 ± 12.71^a^ (64.19–97.31)
MRT (h)	4.37 ± 0.89^a^ (3.10–5.79)	25.66 ± 2.35^c^ (21.94–28.40)	13.41 ± 1.53^b^ (11.09–15.78)
Vz_F_obs	–	8.61 ± 4.40 (6.17–18.30)	3.82 ± 0.82 (2.76–4.8)
Cl_F_obs	–	0.20 ± 0.02 (0.19–0.23)	0.25 ± 0.03 (0.20–0.30)
V_z_obs_	3.06 ± 2.03 (1.52–7.60)	–	–
Cl_obs	0.18 ± 0.05 (0.13–0.30)	–	–
Vss_obs	0.88 ± 0.29 0.56–1.43)	–	–
*F* (%)	–	83.15	69.71

*^a,b,c^Means within the same rows with different letters are statistically significant (*p* < 0.05). *C*_max_, peak plasma concentration; *T*_max_, time to reach peak plasma concentration; AUC, area under the curve from time 0 to the last detectable concentration; MRT, mean residence time; Vss_obs, observed volume of distribution at steady state; Cl_obs, observed clearance from plasma; Vz_F_obs, volume of distribution; *F*, bioavailability; C_0_, initial concentration; C_last_, last measurable concentration; T_1/2λz_, apparent elimination half-life*.

Oxytetracycline LA (IM) compared to the OTC conventional formulations (IM and IV) showed statistically significant differences (*p* < 0.05) (Table 1). OTC goats in Group 2 (LA)’s half-life (*T*_1/2_: 27.96 ± 11.66 h) were significantly longer than the other Groups (*p* < 0.05). In terms of half-life, OTC IV (10.84 ± 3:20 h) and IM (10.47 ± 1.30 h) applications did not show any significant differences statistically (Table 1). Peak plasma concentration (*C*_max_) in goats treated with OTC *via* the IM route (13.57 ± 5.83 μg/ml) and OTC (LA), IM (8.72 ± 2.47 μg/ml) was shown in Table 1. The *T*_max_ was higher (*p* < 0.05) in Group 2 compared to other two Groups (Group 1 *T*_max_: 0.28 ± 0.09 h; Group 3 *T*_max_: 0.46 ± 0.09 h) (Table 1). The latest time that drug was determined plasma was (*T*_last_) 58.28 ± 4.53 h in Group 1, 89.14 ± 11.71 h in Group 2, and 70.29 ± 4.54 h in Group 3. OTC concentration in plasma stayed the longest for Group 3 application (Table 1). The area under the curve (AUC) was much higher (*p* < 0.05) in Group 1 compared to Group 2 and 3 (Table 1). Bioavailability of OTC LA (IM route) and conventional OTC (IM route) at 20 mg/kg dose were 83.15 and 69.71% (Table 1). The plasma OTC concentrations of some animals were not been able to be determined (one animal for OTC IV at 12th hour; and two for IM applications; 12th and 8th hour). Following 48th hour of OTC LA IM administration, no drug was detected in the plasma.

**Figure 1:**
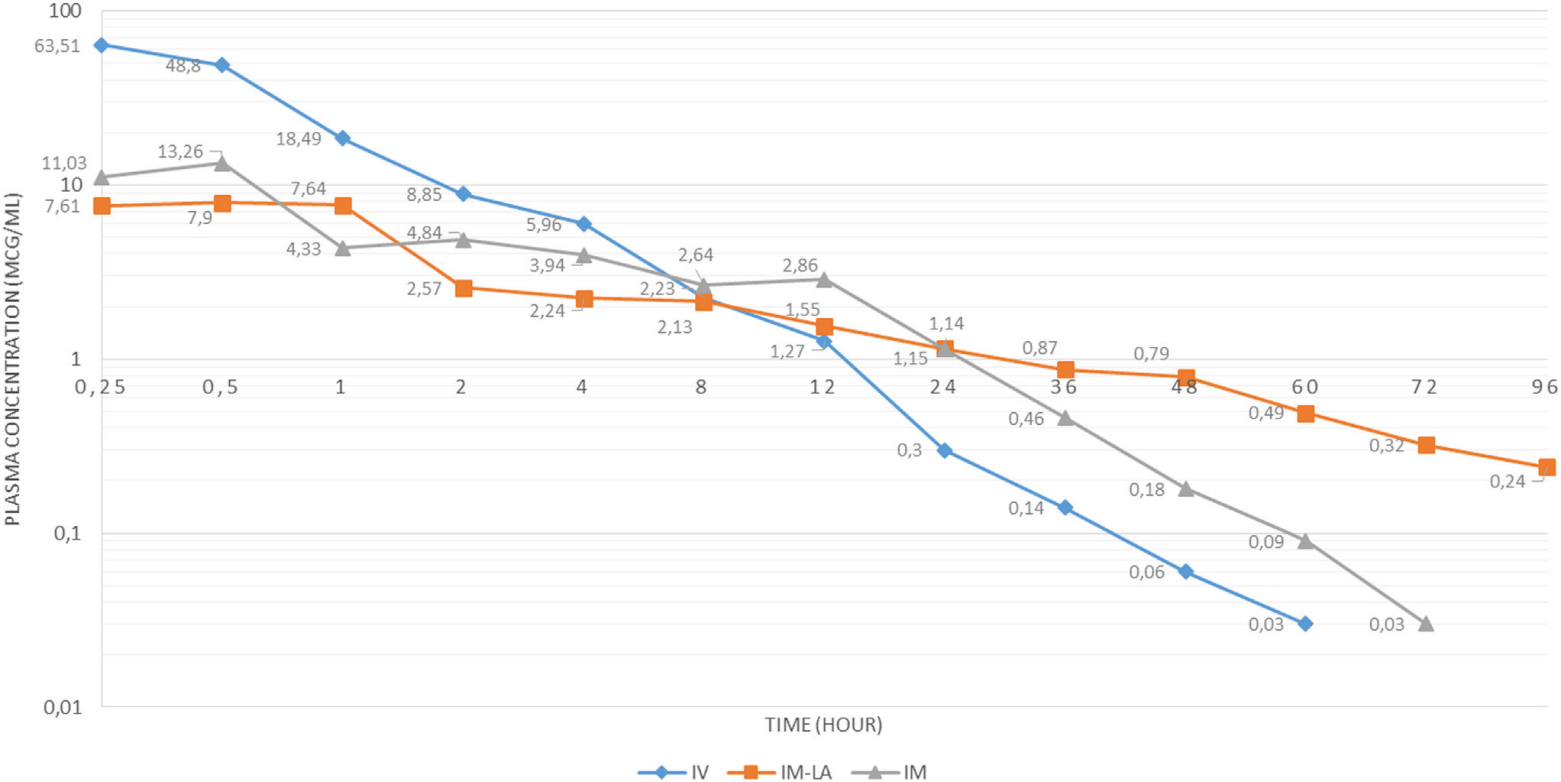
Plasma concentration-time curve for oxytetracycline (OTC) intravenous (IV), OTC long-acting (LA) intramuscular (IM), and OTC IM following administration of 20 mg/kg dose in Kilis Goat.

Plasma concentrations of OTC and OTC LA is given in Figure 1.

## Discussion

Plasma concentrations at different time points following a conventional OTC formulation *via* IV route were homogeneous. The results of conventional OTC IV administered goats showed similar values with Kaya et al. (15) study of sheep administered by conventional OTC IV.

In a pharmacokinetic study with goats that is given 10 mg/kg dose IV, the effective plasma concentration of the conventional oxytetracycline were reached at fourth hour and the Co value was measured as 34.50 ± 1.65 μg/ml. In the study by Mandal et al. (22), half-life (*t*_1/2β_) were found as 1.11 ± 0.05 h, with area under the curve (AUC) 27.69 ± 3.01 μg/ml/h, Vc 0.28 ± 0.03 l/kg, Vd_area_ 0.59 ± 0.06 l/kg, steady state volume of distribution Vd_ss_ 0.51 ± 0.05 l/kg, body clearance Cl 369.67 ± 35.21 ml/kg/h. In the Nubian goats after 5 mg/kg dose of conventional OTC IV administration, the pharmacokinetic parameters were calculated as the half-life *t*_1/2β_ 3.99 h, the area under the curve AUC 8.23 ± 0.08 μg/ml/h, Vd_ss_ 3088 ± 491.50 ml/kg, and Cl_total_ 608.18 ± 5.96 ml/kg/h (23). Elsheikh et al. (23) and Mandal et al. (22) indicated that the possible reason for the differences between pharmacokinetic parameters were the difference in the application concentrations. The correlation between the concentration and the kinetic parameters are seen in these studies along with our study.

In a study carried out on camels, sheep, and goats; OTC was administered at 5 mg/kg IV, where the calculated kinetic parameters, respectively, are, *t*_1/2β_, 2.8, 3.4, and 3.2 h, *C*_max_ 10.2, 850, and 780 µg/ml, Vd_area_ 1.41, 13.4, and 12.1 l/kg. *t*_1/2_ and Vd values for goat and sheep were similar (24). In another study, OTC at 5 mg/kg IV resulted with *t*_1/2β_ as 3.89 and 6.30 h for Nubian goats and desert sheep, AUC 12.08 ± 1.50 and 18.37 ± 1.68 μg/ml/h, Vd_area_ 2.53 ± 0.29 and 2.67 ± 0.39 L/kg, CI 436.99 ± 47.94 and 281.31 ± 25.01 ml/kg/h, respectively. Pharmacokinetic differences of OTC for sheep and goat are thought to be caused by protein bindings and changes in the renal extractions (25). The differences in terms of kinetic parameters with studies of Al-Nazawi (24) and Elsheikh et al. (25) are related to the applied concentration differences; while a correlation is evident.

In the study by Payne et al. (18), OTC LA was administered to goats by IV route (a single dose of 20 mg/kg; Liquamyc L-200); where the plasma density reached to the peak at 0.77 ± 0.83 h (*t*_max_) and *C*_max_ was measured as 8.59 ± 7,47 μg/ml. *T*_1/2_ value was calculated as 14.4 ± 4.92 h, AUC as 67.4 ± 21.7 μg/ml/h, Vd_ss_ as 4.10 ± 1.65 l/kg, and Cl as 0.333 ± 0.117 l/kg/h. The pharmacokinetic parameters, in the current study, were found to be similar to the study by Payne et al (18).

In another study (10), the same OTC LA preparations (Liquamyc L-200) were administered to calves and sheep through IM route at 20 mg/kg; where the *t*_max_ was 3.5 ± 1.2 h, and average *C*_peak_ was 6.1 ± 1.3 μg/ml in sheep. AUC for calves (168 ± 14.6 μg/ml/h) was found to be significantly lower than that for sheep (209 ± 43 μg/ml/h). Vd_ss_ was 3.3 ± 0.49 and 3.08 ± 0.82 l/kg and Cl was 1.88 ± 0.12 and 1.65 ± 0.30 ml/min/kg for calves and sheep, respectively. The difference in pharmacokinetic parameters was related to the differences in species. Compared to the current study, Kilis goats were found to have shorter *t*_max_, higher *C*_peak_, and lower AUC values compared to the calf and sheep.

As a good therapeutic option, OTC is widely used by the veterinarians for the treatment of the diseases of Kilis goats. Since the trait differences have an effect for the optimization of the therapeutic doses of the drugs and no data was available for the pharmacokinetic parameters in Kilis goats, this study provided valuable information for the practicioners in the field. As veterinarians should consider diseases, animal welfare, treatment type, time, place, accessibility to the drugs, and access to the patient for the proper drug (OTC) administration, this paper would expected to contribute for the selection of the administration route and the formulation of the drug for the practitioners as well.

Having a long-time effect and long administration intervals, long effective (LA) OTC preparations are considered as convenient options by veterinarians (4–6, 26). IV infusion which should be administered in every 12 h to ensure the effective concentration is difficult in terms of both animals and practitioners. To provide effective plasma concentration and for ease of administration, IM preparations were found more appropriate. Such studies highlight the necessity of pharmacokinetic studies to adjust the therapeutic dose in the target animal.

## Author Contributions

EY conceived the idea of the study and evaluated pharmacokinetic parameters. İA has given drugs for animals, collected data, analyzed drug, and did the statistical analyses. The manuscript was prepared, edited, and approved by both authors.

## Conflict of Interest Statement

The authors declare that the research was conducted in the absence of any commercial or financial relationships that could be construed as a potential conflict of interest.
